# Medikationsmanagement in der häuslichen Pflege – der Medikationsprozess aus der Perspektive von Klient:innen und ihren Angehörigen

**DOI:** 10.1007/s00391-021-01985-6

**Published:** 2021-10-25

**Authors:** Désirée Diedrich, Franziska Zúñiga, Carla Meyer-Massetti

**Affiliations:** 1 Pflegewissenschaft – Nursing Science, Departement Public Health, Medizinische Fakultät, Universität Basel, Basel, Schweiz; 2 Spitex Grenchen, Grenchen, Schweiz; 3 Berner Institut für Hausarztmedizin (BIHAM), Universität Bern, Mittelstraße 43, 3012 Bern, Schweiz; 4 Klinische Pharmazie und Epidemiologie, Departement Pharmazeutische Wissenschaften, Universität Basel, Basel, Schweiz

**Keywords:** Ambulante Pflege, Patienteninvolvierung, Medikationssicherheit, Schnittstellen, Medikationsfehler, Homecare services, Patient involvement, Medication safety, Interfaces of care, Medication errors

## Abstract

**Hintergrund:**

Eine häufige Aufgabe der professionellen häuslichen Pflege ist die Unterstützung betagter, polypharmazierter Klient:innen beim Medikationsmanagment. Die medikamentöse Versorgung ist oft komplex and anfällig für Medikationsfehler. Aus der Perspektive von Fachpersonen sind insbesondere die zahlreichen interprofessionellen Schnittstellen im Medikationsprozess sowie die Integration von Klient:innen und Angehörigen große Herausforderungen.

**Ziel:**

Mit dieser Studie wurde exploriert, wie Klient:innen und deren Angehörige den Medikationsprozess mit Unterstützung der häuslichen Pflege im Hinblick auf die Medikationssicherheit erleben.

**Methode:**

Es wurde ein qualitativer Forschungsansatz gewählt und leitfadengestützte Interviews mit 8 Klient:innen und 5 Angehörigen durchgeführt. Die Daten wurden anhand der thematischen Analyse nach Braun und Clarke ausgewertet.

**Ergebnisse und Diskussion:**

Vor dem Einbezug der häuslichen Pflege stießen Klient:innen im Medikationsmanagement physisch, psychisch und sozial an ihre Grenzen. Sie erlebten mit der Übernahme des Medikationsprozesses durch die häusliche Pflege Entlastung und Sicherheit. Dabei bringen sie dem Fachpersonal großes Vertrauen entgegen und sehen wenig Bedarf, sich selber in Medikationssicherheitsbestrebungen einzubringen, obwohl Gefahrenquellen im Bereich Selbstmedikation, Adhärenz und Schnittstellen vorhanden sind.

**Schlussfolgerungen:**

Mit der Entlastung durch die professionelle Unterstützung sehen sich Klient:innen der häuslichen Pflege und deren Angehörige kaum mehr als aktive Partner:innen im Wahren der Medikationssicherheit. Es braucht ein Augenmerk der Fachpersonen auf die Bereiche Selbstmedikation und Adhärenz sowie eine Unterstützung bei der Nutzung des Medikamentenplans.

**Zusatzmaterial online:**

Zusätzliche Informationen sind in der Online-Version dieses Artikels (10.1007/s00391-021-01985-6) enthalten.

## Abstract (en)

**Background:**

Professional home care nurses are often tasked with the medication management of older, polymedicated clients.

The medication use process is often complex and prone to medication errors. From the perspective of professional caregivers, the interprofessional interfaces of care as well as the integration of clients and informal caregivers are a major challenge.

**Aim:**

With this study, we explored the perspective of home care clients and their informal caregivers on the medication use process supported by professional caregivers with a focus on medication safety.

**Method:**

We chose a qualitative research approach, using guideline-based interviews with eight clients and five caregivers. Data were analyzed applying the topical analysis according to Braun and Clarke.

**Results and discussion:**

Before engaging professional home care nurses in the medication use process, clients reached their physical, psychological and social limits. They were relieved when the professional home care organization took care of the overall coordination of the medication use process and felt safe. They trust professional caregivers and see limited need to be preoccupied with medication safety themselves, despite risks inherent in self-medication, adherence and transition of care.

**Conclusion:**

Through the relief provided by professional caregivers, home care clients and their informal caregivers no longer perceive themselves as active partners in maintaining medication safety. Healthcare professionals should keep an eye on self-medication as well as adherence and support the use of the medication plan.

## Medikationssicherheit in der häuslichen Pflege

Klient:innen, die zu Hause von ambulanten professionellen Pflegediensten betreut werden, sind meistens betagt und polypharmaziert mit fünf oder mehr Medikamenten [1] und dadurch besonders vulnerabel für medikationsassoziierte Probleme, wie unerwünschte Arzneimittelereignisse und Medikationsfehler („medication errors“ [ME]) [2]. Zahlreiche Schnittstellen machen den Medikationsprozess komplex [3].

**Figure 1:**
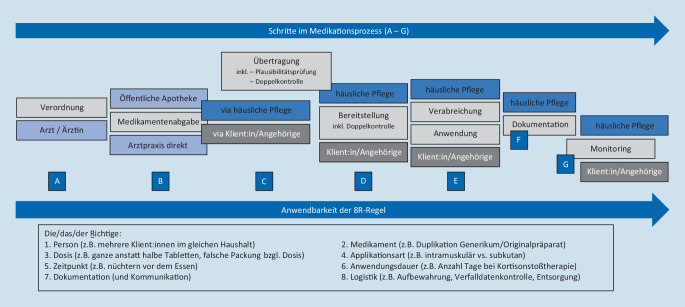

Medikationsassoziierte Probleme treten in der häuslichen Pflege häufiger auf als im stationären Setting [4]. In einer australischen Studie waren 41 % der Klient:innen von ME betroffen [5]. Sie betreffen alle Teile des Medikationsprozesses (Abb. 1): die Verordnung, Beschaffung, Zubereitung, Verabreichung, Dokumentation und Überwachung. Die häufigsten ME daheim sind Verordnungs- und Übertragungsfehler, lückenhafte oder fehlende Dokumentation sowie Fehler beim Bereitstellen, beim Verabreichen und beim Einnehmen [5–7]. Die Verordnung von potenziell inadäquaten Medikamenten betraf in einer Studie bis zu 48 % der Klient:innen [4]. In den USA erhielten 54,5 % der Klient:innen ein falsches Medikament, 31,8 % eine falsche Dosierung, und 13,6 % nahmen Medikamente zum falschen Zeitpunkt ein [8]. Die häufigsten Ursachen für ME sind gemäß Fachpersonen der häuslichen Pflege unzureichende interprofessionelle Kommunikation, fehlende/unklare ärztliche Verordnungen, Übertragungsfehler von Verordnungen sowie Ablenkung beim Richten und beim Verabreichen der Medikamente [3, 5–7]. Gemäß Klient:innen und Angehörigen führen ungenügende Informationen zur Medikation bei Spitalaustritt, ihr mangelndes Wissen bezüglich Medikation sowie unklare, widersprüchliche ärztliche Verordnungen zu ME in bis zu 41 % der Klient:innen [3, 5, 8, 9].

Die aktive Involvierung von Klient:innen in die Entscheidungsfindung und das Management chronischer Erkrankungen ist zentral für eine klientenorientierte Behandlung [10–12]. Bisher ist jedoch wenig zur Sicht von Klient:innen und Angehörigen bekannt. Diese Studie will aufzeigen, wie Klient:innen und Angehörige, die beim Medikationsmanagement durch die häusliche Pflege unterstützt werden, den Medikationsprozess erleben und wo sie ihre Rolle bezüglich der Medikationssicherheit sehen.

## Methode

Für diese Studie wählten wir ein qualitatives Design mit Thornes Methodologie der interpretierenden Beschreibung [13]. Die Daten wurden gemäß der thematischen Analyse nach Braun und Clarke ausgewertet [14]. Damit lassen sich Muster in den Daten identifizieren, analysieren, beschreiben und die Daten interpretieren.

### Untersuchungsort und -gruppe

Die Studie wurde bei einer professionellen häuslichen Non-Profit-Pflegeorganisation im städtischen Umfeld in der Schweiz durchgeführt. Ungefähr 400 Klient:innen/Monat, die von rund 350 Ärzt:innen sowie 2 Krankenhäusern zugewiesen werden, erhalten Unterstützung beim Medikationsmanagement (www.spitex-luzern.ch).

In die Studie schlossen wir Klient:innen ein, die Unterstützung im Medikationsprozess durch Mitarbeitende der häuslichen Pflege erhielten, ≥64 Jahre alt waren und ≥4 ärztlich verordnete Medikamente anwendeten (Zusatzmaterial online: Anhang A). Angehörige schlossen wir ein, wenn sie die Klient:innen im Alltag beim Medikationsprozess unterstützten (Zusatzmaterial online: Anhang B) und das Einverständnis der Klient:innen vorlag. Wir achteten auf Heterogenität im Sample hinsichtlich Geschlecht, Pflegeabhängigkeit sowie Grad der Unterstützung durch Angehörige. Alle mussten Deutsch sprechen, lesen und verstehen können. Die Rekrutierung, inklusive schriftlicher Einwilligung, erfolgte über die häusliche Pflegeorganisation. Die Studie wurde durch die Ethikkommission Nordwest- und Zentralschweiz bewilligt (Projekt-ID: 2018-01225).

### Datensammlung

Die Datensammlung dauerte von August 2018 bis Januar 2019. Die Erstautorin führte anhand eines Leitfadens entweder je ein Einzelinterview mit Klient:innen oder Angehörigen oder Interviews mit beiden gleichzeitig durch (Leitfaden Klient:innen-Interview im Zusatzmaterial online: Anhang C).

Das Interview begann mit der Frage: „Können Sie mir erzählen, wie es dazu kam, dass Sie die Unterstützung der häuslichen Pflege für Ihre Medikamente benötigen?“ Der halbstrukturierte Leitfaden fragte nach den Erfahrungen mit den Teilschritten im Medikationsprozess und danach, wie Klient:innen und Angehörige aktiv zur Verbesserung der Medikationssicherheit beitragen könnten. Der Medikationsprozess im „home care setting“ ist in Abb. 1 abgebildet.

Am Schluss des Interviews wurden soziodemografische Daten erhoben.

Die Interviews – bei den Teilnehmenden zu Hause auf Schweizerdeutsch geführt – dauerten 25–60 min (Mittelwert = 42 min). Sie wurden von Feldnotizen begleitet, digital aufgezeichnet, in Schriftsprache transkribiert und pseudonymisiert.

### Datenanalyse

Die Datensammlung und -analyse erfolgten nach Durchführung des ersten Interviews parallel, um neue Themen in nachfolgenden Interviews explorieren zu können. Die Datenanalyse verlief iterativ und erfolgte gemäß den 6 Schritten der thematischen Analyse nach Braun und Clarke [14]. Für die Datenanalyse verwendeten wir die Software ATLAS.ti® (ATLAS.ti Scientific Development GmbH, Berlin, Deutschland). Der Forschungsprozesses wurde in Seminaren im Rahmen des Masterstudiums in Pflegewissenschaft an der Universität Basel mit Peers kritisch reflektiert.

## Ergebnisse

Es nahmen 13 Personen an der Studie teil: 8 Klient:innen (67- bis 90-jährig), sowie 5 Angehörige (55- bis 76-jährig) (Zusatzmaterial online: Anhänge A und B). Die Ergebnisse zeigten auf, wie Klient:innen und Angehörige mit dem Medikationsmanagement an ihre Grenzen stießen und deshalb die Unterstützung der häuslichen Pflege beanspruchten. Sie erlebten die professionelle Unterstützung im Medikationsprozess als Entlastung und vertrauten dem Fachpersonal, sodass sie die Medikationssicherheit in deren Hände gaben.

### An Grenzen stoßen

Die Mehrheit der Klient:innen hatte die häusliche Pflege involviert, da sie beim selbständigen Medikationsmanagement, insbesondere beim Bereitstellen der Medikamente, physisch, psychisch oder sozial an ihre Grenzen stießen. Eine Klientin berichtete, wie die zunehmende Anzahl an Medikamenten den Überblick für die *Bereitstellung *erschwerte, akzentuiert durch ihre körperlichen Einschränkungen und mangelnde Unterstützung durch Angehörige. Sie erzählte:

Also, bis im Juni hat mein Mann gelebt und hat dies hauptsächlich gemanagt. Und dann habe ich versucht, es selber zu machen. Also das Rausdrücken aus der Folie, das konnte ich kaum, und wenn sie raus ist, ist sie davon gespickt. Für eine Woche bereitstellen, habe ich eine Stunde gebraucht und dann noch auf dem Boden die Tabletten zusammensammeln. (P5)

Das „An-Grenzen-Stoßen“ führte schlussendlich zum Einbezug der häuslichen Pflege.

Angehörige erlebten Grenzen, wenn Klient:innen Medikamente widerwillig oder aufgrund kognitiver Einschränkungen falsch einnahmen sowie deren Einnahme ganz vergaßen. Eine Ehefrau erzählte:

Er hat eine ganz große Abneigung gegen Medikamente, und ich konnte ihn nicht überzeugen, dass er sie nimmt (…). Ich habe einfach diese Verantwortung nicht mehr haben wollen und wollte auch nicht mehr mit ihm streiten … (A9)

Auch die *Logistik der Medikamente* zu Hause, inkl. korrekter Entsorgung, war eine Herausforderung. Der Überblick über bereits vorhandene Medikamente ging verloren, erschwert durch stets neue, zusätzliche Medikamentenverordnungen.

Der Überblick über den aktuellen *Medikamentenplan* war nicht immer gegeben. Eine Tochter erzählte, dass ihre Mutter bis zu 3 verschiedene Medikamentenpläne zu Hause hatte und sie nie wussten, welcher aktuell war. Mit der zunehmenden Menge an Informationen und Schnittstellen waren die Studienteilnehmenden den koordinativen Aufgaben nicht mehr gewachsen und holten Hilfe.

### Entlastung beim Medikationsprozess erleben

Klient:innen und Angehörige waren froh, dass Pflegefachpersonen die Gesamtverantwortung für den Medikationsprozess übernahmen. Das Fachpersonal brachte Struktur und Entlastung in die verschiedenen Prozessschritte, was in Beruhigung und Entspannung resultierte. Die *Bestellung der Medikamente* erfolgte entweder durch die Klient:innen selber oder durch das Pflegepersonal. Als unnötige Belastung erlebten die Klient:innen und/oder ihre Angehörige, dass sie die eigentliche *Besorgung* der Medikamente – d. h. den Transport von der Arztpraxis oder der Apotheke in das Zuhause der Klient:innen – nach der Bestellung selber übernehmen mussten; dies fällt nicht unter die verrechenbaren Leistungen einer häuslichen Pflegeorganisation in der Schweiz, sodass sie hier keine Entlastung bieten konnte.

Die *Bereitstellung der Medikamente *erfolgt entweder durch Mitarbeitende der häuslichen Pflege oder durch Angehörige in einem Mehrwegmedikamentendispenser. In manchen Situationen wird zur Gewährleistung der Medikationssicherheit Hand in Hand gearbeitet: Die Angehörigen stellen die Medikamente bereit und die Mitarbeitenden der häuslichen Pflege kontrollieren die *Anwendung* oder umgekehrt. Das „Vier-Augen-Prinzip“ wird sehr geschätzt. Eine Tochter erzählte:

Die Spitex hat es bemerkt, als ich sie bereitgestellt hatte, dass ich statt einer halben eine ganze Tablette bereitgestellt hatte. (A2)

Das Wissen, dass Angehörige eine sichere Medikation erhalten, war den Interviewten wichtig.

Angehörige berichteten zudem über einen verbesserten *Informationsfluss an Schnittstellen *durch die Involvierung der häuslichen Pflege, da die Pflegefachperson beispielsweise einen aktuellen und vollständigen Medikamentenplan führte, der bei einem Krankenhauseintritt mitgegeben werden konnte.

### Dem Fachpersonal vertrauen und die Medikationssicherheit delegieren

Die Studienteilnehmenden berichteten über ihr großes Vertrauen in die häusliche Pflege. Das Vertrauen kam unterschiedliche zustande: einerseits zeigten die Befragten ein Grundvertrauen, oftmals verbunden mit der Erwartung, dass Mitarbeitende über die notwendige fachliche Kompetenz verfügen. Eine Tochter erzählte:

Dass sie das seriös bereitstellen (…), das ist deren Beruf, also gehe ich davon aus, dass sie es recht machen (…) ein gewisses Grundvertrauen muss man haben. (A10)

Die Interviewten gingen davon aus, dass das Personal seine Arbeit mit der erforderlichen Sorgfalt und Kompetenz ausübt. Andererseits wurde die Vertrauensbildung durch die langjährige Beziehung und positive Erfahrungen mit den Mitarbeitenden unterstützt.

Das Vertrauen zeigte sich sowohl gegenüber dem Pflegefachpersonal wie dem Hausarzt/der Hausärztin. So wussten die Klient:innen und Angehörigen nur wenig über die *verordnete Medikation*; manche kannten die Medikamente gar nicht. Beide Gruppen störten sich jedoch nicht daran. Bei Bedarf wüssten sie sich durchaus Informationen zu beschaffen: via Hausarzt/Hausärztin, andere Angehörige, Beipackzettel oder Medikamentenplan, was jedoch selten genutzt wurde. Sie vertrauten den Hausärzt:innen und dem Pflegefachpersonal, dass die Medikation ihrer Situation angepasst war und korrekt bereitgestellt wurde. Mehrere Klient:innen verfügten weder über einen *Medikamentenplan* noch vermissten sie ihn. Aus ihrer Sicht ist dieser v. a. für Fachpersonen und deren Austausch untereinander gedacht. Angehörige schätzten den Medikamentenplan eher und trugen ihn persönlich bei sich. Insgesamt berichteten die Interviewten kaum über *ME*, weder in der Zusammenarbeit mit professionell Pflegenden noch über eigene. Ein Klient hatte durch das Kontrollieren der Anzahl der Tabletten einmal einen Fehler beim Richten der Medikamente bemerkt und dem Pflegepersonal gemeldet. Er erzählte, wie er mit einem Fehler in Zukunft umgehen würde:

Da schaue ich, wenn ich sie am Morgen herausnehme, da müssen 6 Tabletten darin sein. Wenn jetzt eine fehlen würde, würde ich schauen, was am anderen Tag bereitgestellt ist, und würde diese selber aus der Packung herausnehmen. (P6)

Er wusste sich zu helfen und fühlte sich dadurch sicher.

Klient:innen erzählten, dass sie auch schon die Medikamenteneinnahme wegen häuslicher Abwesenheit ausließen. Dies wird von den Klient:innen als unproblematisch eingestuft: Sie nahmen die vergessene Dosis später ein oder ließen sie weg. Nur wenige besprachen die unterlassene Medikation mit einer Fachperson. Ähnlich unproblematisch wird die Selbstmedikation mit frei verkäuflichen Medikamenten betrachtet. So war den Interviewten der Einfluss von Interaktionen auf die Medikationssicherheit kaum bewusst und sie besprachen die Selbstmedikation nicht mit dem Fachpersonal.

Aus den Erzählungen der Klient:innen und Angehörigen wurde deutlich, dass sie sich bezüglich der Medikation und dem Medikationsprozess sicher fühlten. Grundsätzlich sei gut, was die häusliche Pflege mache. Sie übernehmen dort, wo sie können und entsprechend ihren Kenntnissen durchaus eine Kontrollfunktion und korrigieren ME, doch sie sehen sich nicht als aktive Partner im Gewähren der Medikationssicherheit, sondern sind froh, dass diese „in guten Händen“ ist.

## Diskussion

Der Medikationsprozess bei Klient:innen, die zu Hause von ambulanten professionellen Pflegediensten betreut werden, ist oftmals komplex, nicht zuletzt bedingt durch Polypharmazie und zahlreiche Schnittstellen. Klient:innen der häuslichen Pflege in der Schweiz werden im Durchschnitt mit 16 verordneten Medikamenten (verschiedenen Präparaten) pro Tag therapiert; 87,3 % gelten als polypharmaziert [1]. Akzentuiert wird die Komplexität durch die Schnittstellen unter Leistungserbringern [3, 15], die alle Beteiligten gleichermaßen fordern. Die häusliche Pflege übernimmt oft Aufgaben im Medikationsprozess, wenn Klient:innen und Angehörige an ihre Grenzen stoßen und Verantwortung abgeben möchten. Durch die Unterstützung fühlen sich die Interviewten sicher. Das Vertrauen in die Mitarbeitenden der professionellen häuslichen Pflege ist dabei ein zentrales Element der Delegationsbereitschaft.

Klient:innen sind grundsätzlich an einer korrekten Medikamenteneinnahme interessiert. Die Review von Mira et al. zeigt auf, dass bei älteren, multimorbiden Personen, die ihre Medikamente ohne professionelle Unterstützung bereitstellten, ein 19–59 % höheres Risiko für ME bestand als bei einer Bereitstellung durch Fachpersonen [16]. Auch wenn Klient:innen Medikationssicherheitsprobleme nicht unmittelbar wahrnahmen, leisteten sie einen Beitrag, indem sie professionelle Hilfe beanspruchten. Sie nahmen im Rahmen ihrer Möglichkeiten auch Aufgaben wahr. Doch grundsätzlich lag für die Interviewten die Medikationssicherheit in der Verantwortung der häuslichen Pflege.

Medikationsfehler machen 30–50 % aller Fehler im Gesundheitswesen aus und treten v. a. bei älteren, multimorbiden Personen mit Polymedikation auf [16, 17]. Auch wenn wir mit einer kleinen Stichprobe arbeiteten, ist auffallend, dass ME durchwegs nicht als Thema wahrgenommen wurden. Die Diskrepanz zwischen Literatur und Aussagen der Interviewpartner könnte daran liegen, dass ME nicht erkannt, rechtzeitig identifiziert („near misses“) oder schlicht nicht kommuniziert werden. Möglicherweise nehmen Klient:innen und Angehörige Risiken für die Medikationssicherheit nicht als solche wahr. Ein Risikobereich ist die Selbstmedikation mit frei erhältlichen Medikamenten. Den Klient:innen und Angehörigen war kaum bewusst, dass eine entsprechende Information des Fachpersonals wichtig wäre [18, 19]. Mangelnde Adhärenz, ein Risiko für den Therapieerfolg, wurde ebenfalls kaum mit den Fachpersonen thematisiert [11]. Ebenso wurde von den Klient:innen die Wichtigkeit des Medikamentenplans zur Verbesserung des Informationsflusses an Schnittstellen verkannt, begründet u. a. in der schlechten Verständlichkeit und der verbesserungswürdigen Gestaltung. Aus der Literatur ist jedoch bekannt, dass bereits das Führen und Bei-sich-Tragen eines aktuellen und vollständigen Medikamentenplans das Potenzial hat, die Medikationssicherheit zu verbessern [20, 21].

Gemäß der Literatur ist die aktive Involvierung von Klient:innen und Angehörigen in den Medikationsprozess für die Medikationssicherheit essenziell [22–24]. So nimmt durch eine gezielte Edukation das Wissen zu ihrer Medikation zu, Klient:innen führen einen aktuellen, vollständigen Medikamentenplan, und die Adhärenz wird verbessert, was zur Reduktion von medikationsassoziierten Problemen sowie Spitalwiedereintritten führen kann [10–12]. Viele Interviewte wollten sich jedoch bewusst nicht mehr aktiv einbringen, nachdem sie die Verantwortung einmal in professionelle Hände gelegt hatten.

Obwohl im Lauf der Interviews eine Datensättigung erreicht wurde, weist die Studie verschiedene Limitationen auf. So nahmen auf Basis der gezielten Rekrutierung möglicherweise eher kollaborative und eloquente Klient:innen und Angehörige teil. Ebenso handelt es sich um Teilnehmende einer Organisation der häuslichen Pflege in einer Region mit Selbstdispensation, also direkter Abgabe von Medikamenten durch Ärzt:innen. Dieses in Europa einmalige Konzept eliminiert oftmals die Apotheken aus dem Medikationsprozess, was insbesondere dem Vier-Augen-Prinzip und der Klient:innenedukation abträglich sein kann. Diese Besonderheit muss bei der Übertragung der Resultate in andere Settings berücksichtigt werden.

## Fazit für die Praxis

Der Medikationsprozess im Setting der häuslichen Pflege ist aufgrund der Polymedikation und vieler Schnittstellen komplex. Klient:innen und Angehörige empfinden den Medikationsprozess aufgrund der Unterstützung durch die professionelle häusliche Pflege jedoch als sicher.

Aus den Erzählungen der Klient:innen und Angehörigen zeigen sich folgende Punkte, wo das Fachpersonal sie gezielt involvieren kann:

- Die Selbstmedikation wird kaum mit dem Hausarzt/der Hausärztin besprochen. Deren Thematisierung kann die Sicherheit in Kombination mit der verordneten Arzneimitteltherapie gewährleisten.
- Die Adhärenz ist zentral für den Therapieerfolg. Die Unterstützung bei der Medikation sowie das Ansprechen von Lücken unterstützen den Therapieerfolg.
- Die Verwendung einer aktuellen, kompletten, korrekten, gut verständlichen Medikamentenliste optimiert die Medikationssicherheit an Schnittstellen und soll gezielt gefördert werden.

## Supplementary Information






