# Supplementary material for: Medication management in home care—The medication use process from the perspective of clients and their caregivers
Source: Z Gerontol Geriatr. 2021 Oct 25;55(8):667–72. [Article in German] doi: 10.1007/s00391-021-01985-6 (PMC9726664; doi:10.1007/s00391-021-01985-6)
Supplement: Supplementary file 1 [file 391_2021_1985_MOESM1_ESM.docx]

Anhang A: Soziodemographische und klinische Daten der Klient:innen

| **Teilnehmende** (n) | **8** |  |
| --- | --- | --- |
| **Geschlecht:**  Frauen  Männer | 4  4 |  |
| **Alter:** (Jahre) | **67-90** |  |
| **Zivilstand:**  verheiratet/ Partnerschaft  verwitwet  ledig  geschieden, getrennt | 1  3  2  2 |  |
| **Wohnform:**  alleine  mit Partner:in | 7  1 |  |
| **Bildungsabschluss:**  Hochschule  Höhere Berufsbildung  berufliche Grundbildung  Andere | 1  2  2  3 |  |
| **Berufsbranchen:**  Bildung und Unterricht  Fahrzeuge  Logistik  Medien: Druck, Verlag, Dokumentation  keinen Beruf erlernt | 3  1  1  1  2 |  |
| Anzahl Jahre **Unterstützung** durch Mitarbeitende der Spitex Stadt Luzern | 0.3-10 |  |
| Anzahl **Haupt-Diagnosen** | 3-9 |  |
| Anzahl ärztlich verordnete **Dauermedikamente** | 6-13 |  |
| Anzahl ärztlich verordnete **Reservemedikamente** | 1-6 |  |
| **Unterstützung im Medikationsprozess durch Angehörige (A) und/oder Mitarbeitende der Spitex (S):**  Wegbegleitung zu Arzttermin  Teilnahme an Arzttermin  Anleiten/Hilfestellung Besorgung Medikamente  Besorgung Medikamente  Anleiten/Hilfestellung Medikamente bereitstellen  Medikamente bereitstellen  Anleiten/Hilfestellung Medikamenteneinnahme  Anleiten/Hilfestellung Wirkung und Nebenwirkung  Überwachung Wirkung und Nebenwirkung  Anleiten/Hilfestellung Medikamentenplan führen  Führen des Medikamentenplans  Anleiten/Hilfestellung Spitex Leistungen organisieren  Spitex Leistungen organisieren | A  3  2  1  3  1  2  2  3  0  2  1  3  1 | S  0  0  5  0  1  6  2  4  1  2  5  1  - |
