# Supplementary material for: Medication management in home care—The medication use process from the perspective of clients and their caregivers
Source: Z Gerontol Geriatr. 2021 Oct 25;55(8):667–72. [Article in German] doi: 10.1007/s00391-021-01985-6 (PMC9726664; doi:10.1007/s00391-021-01985-6)
Supplement: Supplementary file 2 [file 391_2021_1985_MOESM2_ESM.docx]

Anhang B: Soziodemographische Daten der Angehörigen

| **Teilnehmende (n)** | **5** |
| --- | --- |
| **Geschlecht:**  Frauen  Männer | 5  0 |
| **Beziehung zu Patient/-innen:**  Ehefrau  Tochter  Schwester | 1  3  1 |
| **Alter:** (Jahre) | 55-76 |
| **Zivilstand:**  Verheiratet / Partnerschaft  ledig  geschieden, getrennt | 2  2  1 |
| **Bildungsabschluss:**  Höhere Berufsbildung  berufliche Grundbildung | 2  3 |
| **Berufsbranchen:**  Banken und Versicherungen  Bühne | 4  1 |
| **Unterstützung im Medikationsprozess:**  Wegbegleitung zu Arzttermin  Teilnahme an Arzttermin  Anleiten/Hilfestellung Besorgung Medikamente  Besorgung Medikamente  Anleiten/Hilfestellung Medikamente bereitstellen  Medikamente bereitstellen  Anleiten/Hilfestellung Medikamenteneinnahme  Anleiten/Hilfestellung Wirkung und Nebenwirkung  Überwachung Wirkung und Nebenwirkung  Anleiten/Hilfestellung Medikamentenplan führen  Führen des Medikamentenplans  Anleiten/Hilfestellung Spitex Leistungen organisieren  Spitex Leistungen organisieren | 5  3  1  4  1  2  2  1  2  1  3  3  3 |
