# Supplementary material for: Medication management in home care—The medication use process from the perspective of clients and their caregivers
Source: Z Gerontol Geriatr. 2021 Oct 25;55(8):667–72. [Article in German] doi: 10.1007/s00391-021-01985-6 (PMC9726664; doi:10.1007/s00391-021-01985-6)
Supplement: Supplementary file 3 [file 391_2021_1985_MOESM3_ESM.docx]

**INTERVIEWLEITFADEN PATIENT**

**Einleitung**

Um möglichst viele Informationen von den Studienteilnehmenden zu erhalten, werden offene Fragen anhand eines Leitfadens gestellt. Der Leitfaden beinhaltet Fragen zu Erfahrungen rund um ihr Medikationsmanagement mit der Spitex Stadt Luzern und zu ihrer Sicht, wie sie zur Verbesserung der Medikationssicherheit beitragen können. Offene Fragen sollen zum freien Erzählen anregen und mit Vertiefungsfragen werden die angesprochenen Themen weiter exploriert. Der Ablauf der Fragen ergibt sich aus dem Gespräch.

**Vorbereitung des Interviews**

Die Erhebung der soziodemographischen und klinischen Daten der Patienten erfolgt vor dem Interview in der elektronischen Patientenakte Swing ®. Ergänzungen erfolgen durch mündliche Befragung nach dem Interview.

**Ablauf des Interviews**

- ***Begrüssung*** des Studienteilnehmers:
- „*Guten Tag, es freut mich Sie kennen zu lernen.“*
- „*Ich bin Fr. Diedrich, diplomierte Pflegefachfrau, ich studiere Pflegewissenschaft an der Universität in Basel und arbeite in der Spitex Grenchen.“*
- *„Wir haben heute einen Termin für das Interview vereinbart.“*
- „*Vielen Dank, dass Sie bei dieser Studie mitmachen.“*
- *Einrichten für das Interview und die digitale Aufnahme*
- ***Studieninformationen***: *„Ich greife nochmals einige wichtige Punkte aus der Studieninformation auf“:*
- *„Das Interview dauert circa 60 Minuten.“*
- *„Sie dürfen mir jederzeit mitteilen, wenn Sie das Interview unterbrechen oder abbrechen möchten. Wir besprechen dann zusammen, wie wir verbleiben wollen.“*
- *„Es steht Ihnen frei Fragen nicht zu beantworten.“*
- *„Alle Informationen werden vertraulich behandelt, und die Daten werden verschlüsselt, damit keine Rückschlüsse auf Personen möglich sind.“*
- *„Das Interview wird digital aufgezeichnet.“*
- *„Notizen von mir während des Interviews dienen als Gedankenstützen.“*
- „*Haben Sie noch Fragen zur Studie oder zur Durchführung des Interviews?“*

**Einstieg ins Interview**

- *„Mit dieser Studie soll untersucht werden, wie Sie die Zusammenarbeit mit der Spitex Stadt Luzern und den Umgang mit Ihren Medikamenten erleben und wie Sie aus Ihrer Sicht zur Sicherheit im Umgang mit Ihren Medikamenten beitragen können.“*
- *„Ich würde jetzt die digitale Aufnahme starten, ist das ok für Sie?“*

🡪 ***Digitale Aufnahme starten***

| **Leitfragen** | **Spezifische Fragen** | **Vertiefungsfragen** |
| --- | --- | --- |
| **Medikationsprozess**  **Können sie mir erzählen wie es dazu kam, dass Sie die Unterstützung der Spitex für Ihre Medikamente benötigen?**  **Können Sie mir erzählen, wie das bei Ihnen mit den Medikamenten abläuft?**  **Wie sieht Ihr Tagesablaufes, im Umgang mit Ihren Medikamenten aus?** | **Medikationsprozess**   - Was machen Sie **selber** im Umgang mit Ihren **Medikamenten**? Was macht die **Spitex**? - Was geht Ihnen **einfach** / Was finden Sie **schwierig** im Umgang mit Ihren Medikamenten? - Haben Sie schon mal erlebt, dass es zu **Schwierigkeite**n kam? - Nehmen Sie die **Medikamente regelmässig** ein? Einige Patienten haben mir erzählt, dass es auch schon mal vorgekommen ist, dass sie vergessen haben **Medikamente zu nehmen**, ist das bei ihnen auch schon passiert? Wann vergessen sie die Medikamente zu nehmen? - Einige Patienten haben mir erzählt, dass auch schon **Fehler** im Umgang mit Medikamenten passiert waren, ist das bei Ihnen auch schon vorgekommen? Wie war das für Sie, was **bedeutete** das für Sie? - Gibt es etwas, das Sie besonder**s schätzen/Ihnen besonders wichtig** ist, im Zusammenhang mit Ihren Medikamenten?   **Medikamentenplan**   - Haben Sie einen **Medikamentenplan**? Können Sie mir diesen zeigen und **erklären**? - Welche **Informationen** hätten Sie gerne **auf dem Medikamentenplan**? **Wie** sind Sie zu **Informationen** über **Ihre Medikamente** gekommen? Wer hat Ihnen die Medikamente erklärt? **Was** für **Informationen** über Ihre **Medikamente** würden Sie sich **wünschen**? Von **wem** möchten Sie diese Informationen **erhalten**? - **Wen kontaktieren** Sie bei **Fragen /oder Problemen** zur Ihren **Medikamenten**? Können Sie **beschreiben**, wie sie dabei **vorgehen**? **Was** sind das für **Fragen /oder Probleme** die Sie über **Ihre Medikamente** haben? - **Wer** hält den **Medikamentenplan aktuell**?   **Selbstmedikation**   - Nehmen Sie **Medikamente** ein, die Sie **ohne ein Rezept** des Arztes in der Apotheke kaufen z.B. Vitaminpräparate, Sprays, Tropfen, Salben, Pflaster, pflanzliche Präparate (z.B. Johanniskraut) oder homöopathische Mittel? **Besprechen** Sie das mit dem **Arzt**?   **Bestellung/Besorgung**   - Können sie erzählen, wie das bei Ihnen mit der **Bestellung abläuft**? - Haben Sie es schon einmal erlebt, dass Ihnen **Medikamente gefehlt** haben? Was machen Sie, wenn Ihnen die **Medikamente ausgehen**? | - Können Sie darüber noch etwas mehr erzählen? - Was ist passiert? - Können Sie ... noch ausführlicher beschreiben? - Was haben Sie danach gemacht? - Wie ging es weiter? - Und dann? - Können Sie mir ein konkretes Beispiel nennen? - Können Sie beschreiben wie es dazu kam? - Habe ich das richtig verstanden? - Was ist Ihnen sonst noch aufgefallen? |

| **Kommunikation**  Sie haben nun schon viel erzählt über Probleme und Lösungen mit den Medikamenten. Ein Thema würde mich noch interessieren und das wäre die Kommunikation.  **Erzählen Sie mir doch bitte, wie die Kommunikation bezüglich Medikamenten zwischen Ihnen, dem Hausarzt und der Spitex abläuft? z.B. wenn Sie ein neues Medikament erhalten oder eine Änderung Ihrer Medikation erfolgt** | **Kommunikation**  **Schnittstelle**  **Spitex – Patient – Angehörige - Hausarzt**   - Wenn der Arzt Ihnen ein **neues Medikament verordnet**, wie geht es dann weiter? - Wie zufrieden sind Sie mit diesem **Ablauf**? - Können Sie die **Zusammenarbeit** mit der Spitex und mit Ihrem Hausarzt beschreiben? - Haben Sie schon mal erlebt, dass es zu **Schwierigkeiten** kam?   **Spital – Patient – Angehörige - Spitex**   - Wie werden **Informationen zur Medikation** übermittelt, wenn Sie **ins Spital** müssen? - ...und Sie wieder **nach Hause** kommen? - Sind Sie zufrieden mit diesem **Ablauf**?   **Kommunikation allgemein**   - Gibt es etwas, das Sie besonders **schätzen/wichtig** ist, bezüglich **Kommunikation**? | - Können Sie darüber noch etwas mehr erzählen? - Was ist passiert? - Können Sie ... noch ausführlicher beschreiben? - Was haben Sie danach gemacht? - Wie ging es weiter? - Und dann? - Können Sie mir ein konkretes Beispiel nennen? - Können Sie beschreiben wie es dazu kam? - Habe ich das richtig verstanden? - Was ist Ihnen sonst noch aufgefallen? |
| --- | --- | --- |
| **Interventionen**  Sie haben mir bereits viel erzählt wie es bei Ihnen läuft mit den Medikamenten und wo es Schwierigkeiten gegeben hat.  **Wo sehen Sie Verbesserungsmöglichkeiten im ganzen Umgang mit den Medikamenten?**  **Wie können Sie dazu beitragen?** | **Probleme, Ideen, Verbesserungsvorschläge**   - Können Sie mir von einer Gelegenheit erzählen, wo Sie selber ein **Problem erkannt** und gelöst haben? - Haben Sie mit dem **Spitex** schon mal über ein **Problem bei der Medikation gesprochen**? - Können Sie sich vorstellen bei der **Verbesserung mit der Spitex zusammenzuarbeiten**? **Wie** würde das konkret **aussehen**? | - Was würden Sie sich wünschen? - Was würde Ihnen helfen? - Was müsste geändert/verbessert werden? - Wie könnte es verbessert werden? Haben Sie Vorschläge? - Können Sie ein Beispiel machen? - Wie möchten Sie dabei mithelfen/involviert werden? - Was kommt für Sie nicht in Frage? |
| **Explorierte Fragen** | Mit einigen Personen mit denen ich schon ein Interview geführt habe, beschrieben, dass   - das **Vertrauen** eine Rolle spielt bezüglich Medikationssicherheit, was meinen Sie dazu? - die **Ausbildung des Personals** eine Rolle spielt bezüglich Medikationssicherheit, was meinen Sie dazu? |  |
| **Abschlussfrage** | Gibt es etwas was Sie mir noch sagen möchten? |  |

*„Ich würde jetzt die digitale Aufnahme stoppen, ist das ok für Sie?“*

***🡪 Digitale Aufnahme stoppen***

- ***Fragebogen***: Fehlende soziodemographische und klinische Daten ergänzen. „*Bevor wir das Interview abschliessen, bräuchte ich noch einige spezifische Angaben von Ihnen.“*

**Abschluss des Interviews**

- *„Vielen Dank, dass Sie so offen waren über Ihr Erlebtes zu sprechen, das war sehr interessant.“*
- „Sie werden nach Abschluss der Studie eine schriftliche Zusammenfassung der Ergebnisse erhalten.“
- *„Ihre Teilnahme an der Studie ist jetzt für Sie abgeschlossen.“*
- *„Vielen Dank für Ihre Teilnahme an der Studie und ich wünsche Ihnen noch einen schönen Tag.“*

**Nachbereitung des Interviews**

- Feldnotizen
